# No effect of hippocampal lesions on stimulus-response bindings

**DOI:** 10.1016/j.neuropsychologia.2017.07.024

**Published:** 2017-08

**Authors:** Richard N. Henson, Aidan J. Horner, Andrea Greve, Elisa Cooper, Mariella Gregori, Jon S. Simons, Sharon Erzinçlioğlu, Georgina Browne, Narinder Kapur

**Affiliations:** a MRC Cognition & Brain Sciences Unit, Cambridge, UK; b Department of Psychology, University of York, UK; c Neuropsychology Department, Addenbrooke's Hospital, Cambridge University Hospitals NHS Foundation Trust, UK; d Department of Psychology, University of Cambridge, UK; e Research Department of Clinical, Educational and Health Psychology, University College London, UK

**Keywords:** Hippocampus, Amnesia, Response learning

## Abstract

The hippocampus is believed to be important for rapid learning of arbitrary stimulus-response contingencies, or S-R bindings. In support of this, Schnyer et al. (2006) (Experiment 2) measured priming of reaction times (RTs) to categorise visual objects, and found that patients with medial temporal lobe damage, unlike healthy controls, failed to show evidence of reduced priming when response contingencies were reversed between initial and repeated categorisation of objects (a signature of S-R bindings). We ran a similar though extended object classification task on 6 patients who appear to have selective hippocampal lesions, together with 24 age-matched controls. Unlike Schnyer et al. (2006), we found that reversing response contingencies abolished priming in both controls and patients. Bayes Factors provided no reason to believe that response reversal had less effect on patients than controls. We therefore conclude that it is unlikely that the hippocampus is needed for S-R bindings.

## Highlights

• Hippocampus is thought important for rapid binding of stimuli (S) and responses (R).
• Six patients with hippocampal damage showed evidence of normal S-R bindings.
• Both patients and controls showed priming of object size judgments.
• Patients and controls showed equivalent priming reductions when responses reversed.
• The hippocampus is not necessary for this type of S-R binding.

## 1 Introduction

The medial temporal lobes (MTL), and hippocampus in particular, are thought necessary for rapid acquisition of new associations (Squire, 1992, Cohen and Eichenbaum, 1994, Schacter and Tulving, 1994; Giovanello et al., 2004). On the other hand, such MTL regions do not appear necessary for all types of rapid plasticity, such as that presumed to underlie phenomena like priming, which can also occur after a single exposure to a stimulus (e.g., Cave and Squire, 1992; Schacter et al., 1993). Priming is often measured by decreases in the reaction time (RT) to perform a simple classification task on a stimulus, such as deciding whether the object depicted by a picture is large or small in real life. Such RT priming has often been associated with facilitated perceptual or conceptual processing, occurring in cortical regions outside the MTL (Moscovitch, 1994).

However, recent studies have shown that the dominant cause of such classification-based RT priming is the encoding and retrieval of Stimulus-Response (S-R) bindings (see Henson et al., 2014, for a recent review). According to this account, the response made to the first presentation of a stimulus is bound together with that stimulus, such that when that stimulus is repeated, the response can be retrieved. This retrieval of a previous response is assumed to be faster than repeating the original perceptual/conceptual processing that generated the response on the initial stimulus presentation, causing the RT priming. However, if the task changes between initial and repeated presentations, such that the response is changed, the amount of RT priming is reduced. Indeed, sometimes priming is abolished by a response reversal, or even becomes negative, i.e, slower RTs for repeated than novel stimuli, possibly owing to interference from retrieval of incorrect responses (Horner and Henson, 2011). This difference in the amount of priming as a function of whether or not the response on second presentation is congruent with that on first presentation – the “congruency effect” – is often used as the defining signature of S-R bindings.

Neuroimaging data support the contribution of rapidly learnt S-R bindings to performance on classification tasks. Several fMRI studies in healthy individuals have found that the decreased fMRI response following repetition of visual stimuli (“repetition suppression”, RS), which has been associated with priming (Koutstaal et al., 2001, Schacter and Buckner, 1998, Simons et al., 2003), is reduced when the classification task is reversed. This reduction in RS following response reversal has been seen in lateral prefrontal regions commonly associated with response selection, and occasionally in ventral temporal regions commonly associated with perceptual/conceptual component processes (Dobbins et al., 2004, Horner and Henson, 2008, Race et al., 2009), though is not readily apparent in MTL regions.

Given that a typical priming experiment entails tens if not hundreds of unique stimuli, the retrieval of the appropriate S-R binding when one of those stimuli is repeated suggests that the brain has an impressive capacity to store many such S-R bindings. To test whether this capacity for rapid learning of multiple, unique S-R associations is supported by MTL, Schnyer et al. (2006; Experiment 2) reported priming data from a speeded classification task on nine patients with MTL damage, together with age-matched controls. Participants were initially asked to decide “Is the object bigger than a shoebox?”, but then after one or three presentations of each stimulus, the task reversed to “Is the object smaller than a shoebox?”. Controls showed the usual reduction in RT priming when the task was reversed, indicative of S-R bindings. RT priming in the patients however showed no detectable effect of the task being reversed (see ahead to Fig. 3 for a re-plotting of Schnyer et al.*'*s data). The authors therefore concluded that MTL regions are responsible for S-R learning.

Though MTL damage was “radiologically-verified” in each patient, the extent of that damage was not reported by Schnyer et al. (2006), so they were unable to conclude whether S-R bindings are supported specifically by the hippocampus, or by other MTL regions like entorhinal, perirhinal or parahippocampal cortices. We recently reported six patients whose MRI scans showed clear evidence of hippocampal volume reduction, with little sign of gray-matter damage outside the hippocampus (Henson et al., 2016). Our main aim in the present experiment was therefore to determine whether the S-R deficit reported by Schnyer et al. is specific to hippocampal damage.

**Fig. 1:**
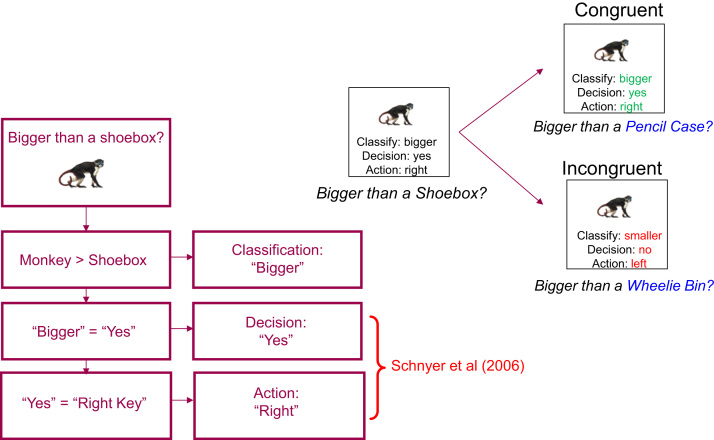
Bottom Left: Schematic of possible response representations (Classifications, Decisions and Actions) that could be bound with a stimulus in a classification task. Reversing the task, e.g., from “Bigger than a shoebox” to “Smaller than a shoebox”, as in Schnyer et al. (2006), reverses the Decision and Action, but not the Classification. Top Right: Changing the referent (e.g, from a shoebox to a wheelie bin), on the other hand, reverses all three levels of response representation.

Our second aim was to test whether Schnyer et al.*'*s results generalise to a modified version of the object classification task. Our modified paradigm (initially proposed by Denkinger and Koutstaal (2009) involves keeping the task constant (e.g, “Is the object bigger than X?”), but changing the referent instead (i.e, X). This paradigm simultaneously reverses all three levels of response representations in S-R bindings that have been identified to date (Horner and Henson, 2011; see also Schnyer et al., 2007; Dennis and Perfect, 2012). This is illustrated in Fig. 1, where the response associated with an object (e.g, monkey) when it is judged to be bigger than a shoebox could include the specific motor Action (e.g, right index finger press), the Decision (e.g, “yes”/“no”) and/or the Classification label (e.g, “bigger”/“smaller”). Reversing the task, as done in the Schnyer et al. paradigm, potentially disrupts the value of retrieving the previous Action and/or Decision (i.e, disrupts S-A and/or S-D bindings), but retrieving the previous Classification label (e.g., “bigger”) could still help generate a response (e.g, “no” to the reversed task of “smaller than a shoebox?”). Note that we use to term “classification” to refer to the binary category label given to the object at Study (e.g., “bigger” or “smaller”); more complex stimulus-task associations, or semantic information about the objects, are also likely to contribute to priming in general, but are kept constant in the current paradigm (see Discussion section for fuller consideration of these issues). Indeed, it is possible that the residual priming in Schnyer et al.’s reversal condition, which they attributed to facilitation of perceptual/conceptual processes outside the MTL, actually reflected intact stimulus-classification (S-C) bindings in their patients (despite impaired S-A and/or S-D bindings). On the other hand, changing the referent, for example to a wheelie bin^1^ (Fig. 1), additionally disrupts the value of retrieving a prior Classification, as shown by Horner and Henson (2009), and may therefore abolish any priming in patients with hippocampal damage.

Furthermore, we can also test the type of stimulus representation in S-R bindings by orthogonally varying whether or not the stimulus is repeated in the same perceptual form (e.g, picture or word) as its initial presentation. We previously showed evidence for two levels of stimulus representation: a form-specific and more abstract representation (Horner and Henson, 2011; see also Allenmark et al., 2015; though see Schnyer et al., 2007). We included this “Within-format” versus “Across-format” manipulation in the present experiment to test whether patients are similarly able to form S-R bindings that abstract away from the precise stimulus form. Indeed, the present experiment is identical to that in Experiment 1 of Horner and Henson (2011), except that we: 1) tested older healthy controls and patients, rather than young controls, 2) made trials self-paced rather than running at a fixed rate, to make the task easier for patients (and older controls), who generally respond slower and show greater variability, and 3) used two rather than three presentations of each stimulus before the referent change, to try to maintain the same total duration as our previous experiment.

More precisely, Experiment 1 conformed to a 2 × 3 × 2 factorial design, with between-subject factor Group (N = 24 Controls vs N = 6 Patients) and within-subject factors: Study Condition (Within-format Primed, Across-format Primed, Novel) and Congruency (Congruent, Incongruent; see Methods section for how Novel trials were split into Congruent and Incongruent conditions). Like Horner and Henson (2011), we defined priming in multiple ways, but focus on the proportional measure ((Novel–Primed)/Novel) used by Schnyer et al. (2006) to allow for the fact that patients tend to have longer overall RTs than controls. Once priming scores have been calculated, the design equates to a 2 (Group) × 2 (Format) × 2 (Congruency) factorial design. Based on Schnyer et al.'s findings, we expected an interaction between Group and Congruency on the amount of priming, with controls showing a greater effect of congruency than patients. More specifically, we predicted that controls would show greater priming than patients in Congruent trials (because controls but not patients benefit from S-R bindings), but comparable or even less priming than patients for Incongruent trials (where controls would either ignore S-R bindings, or experience interference from incompatible S-R bindings).

## 2 Materials and methods

### 2.1 Participants

The six patients were selected from the Cambridge Hippocampal Panel, and are the same as those reported in Henson et al. (2016). The study was approved by NRES Ethics Committee East of England (ref 12/EE/0190) and written consent obtained according to the Declaration of Helsinki. The patients were referred on the basis of reported memory difficulties and, in some cases, a diagnostic MR scan that showed an indication of limited MTL damage.

A summary of the patients is given in Table 1. The Z-scores for verbal memory, visuospatial memory, verbal memory, visuospatial skills and executive function are combined across multiple neuropsychological tests (using Stouffer's method; see Henson et al., 2016, for details and scores of precise tests). All patients were impaired in verbal and/or visual memory: although the combined Z-score was not significant for P5, this was driven by intact recognition memory (75–95th percentile for words; 50th percentile for faces), and when restricted to recall tests, her memory varied from 10 to 25th percentile for stories and 2–25th percentile for complex figures (see Henson et al., 2016). The only non-memory impairment was executive function for P4 (see Supplementary Material for analyses with P4 excluded). All six patients showed significant reduction in hippocampal volume; two showed additional reduction in entorhinal volume (P4 and P6) and two showed additional reduction in parahippocampal volume (P2 and P6). Whole-brain voxel-wise analysis did not reveal any significant group differences from age- and sex-matched controls outside the hippocampus (Henson et al., 2016).

**Table 1:** Summary of patients. Neuropsychological scores are combined Z-values, based on norms; Hippocampal volumes are reported as T-values relative to age- and sex-matched controls (with % of control volume in brackets) from Henson et al. (2016). Executive function was collapsed across digit-symbol, forward digit span, backward digit-span and the Brixton test. For Anxiety/Depression, the “Mild” label is based on HADS score of 9 in both cases; “None” means score < 8. Missing data indicated by hyphen. For further details, see Henson et al. (2016). Parahipp = Parahippocampal. * = p < 0.05, two-tailed; ~ = p < 0.05, one-tailed.

**Patient**	**P1**	**P2**	**P3**	**P4**	**P5**	**P6**
Age (years)	57	39	66	66	57	62
Gender	Male	Male	Male	Male	Female	Male
Education	< 12	14	12+ Apprentice	14+	14+	12+ Apprentice
(years)						
Presenting diagnosis	Limbic encephalitis	Carbon monoxide	Carbon monoxide	Limbic encephalitis	Limbic encephalitis	Limbic encephalitis
History of symptoms (years)	-, 1	20	18	6	6	14, 4
NART IQ	91	112	123	118	121	111
Verbal Memory (Z)	−3.31*	−2.68*	−2.68*	−4.03*	−0.48	−3.12*
Visuospatial Memory (Z)	−3.82*	−2.87*	−2.42*	−3.82*	−1.59	−0.91
Verbal Skills (Z)	−1.37	+0.14	+1.39	+1.44	+0.29	+0.99
Visuospatial Skills (Z)	−0.54	+2.97	+2.40	−0.88	+1.24	+1.45
Executive Function (Z)	−0.76	+1.37	+0.26	−3.12*	+1.21	+0.21
Anxiety / Depression	Mild	–	None	None	None	Mild
Hippocampal	−5.07	−4.81	−3.32	−1.80	−3.95	−4.97
Volume (T)	(46)*	(57)*	(58)*	(79)~	(67)*	(50)*
Entorhinal	−1.76	−1.59	−0.85	−2.93	−0.97	−3.67
Volume (T)	(79)	(79)	(88)	(61)*	(85)	(52)*
Parahipp.	−0.13	−2.52	+1.07	−2.01	+0.18	−3.12
Volume (T)	(98)	(70)*	(112)	(76)	(102)	(62)*

Twenty-four control participants were recruited from the Volunteer Panel of the Medical Research Council (MRC) Cognition and Brain Sciences Unit (CBU). There was no significant difference in the ages of these controls (M = 60, range 50–72) and those of the patients (M = 58, range 39–66), in terms of the mean, t(28) = 0.54, p = 0.60, or the variance, Levene's test, F(1,28) = 1.34, p = 0.26. Thirteen of the control group were female, whereas only one patient was female, and therefore analyses were also repeated with sex as a covariate. These potential confounds were further addressed by reporting tests of each individual patient versus the control group.

### 2.2 Materials

Stimuli were 384 coloured images of everyday objects and their names, previously used by Horner and Henson (2009), split into two groups, relating to the wheelie bin and pencil case referent change (192 stimuli per group). For the wheelie bin referent group, stimuli were classified so that 25% were smaller than both a shoebox and a wheelie bin (Congruent), 50% were bigger than a shoebox but smaller than a wheelie bin (Incongruent) and 25% were bigger than both a shoebox and a wheelie bin (Congruent). For the pencil case referent group, 25% were smaller than a pencil case and a shoebox (Congruent), 50% were bigger than a pencil case but smaller than a shoebox (Incongruent) and 25% were bigger than a pencil case and a shoebox (Congruent). This resulted in 96 stimuli per Congruency condition for each referent group. Stimuli within each of these Congruency groups were randomly assigned to one of three Study Condition groups, relating to whether they were presented as a picture at Study (Within-format Primed), a word at Study (Across-format Primed) or were experimentally Novel (Novel). This resulted in 64 stimuli per each of the 3 conditions when collapsing across the two referent changes. The assignment of stimuli to the three Study Condition factors was rotated across control participants.

### 2.3 Procedure

Prior to the experiment, participants performed a practice session using the “bigger-than-shoebox” task, where it was made clear that this comparison referred to the object's typical size in real life. Participants responded using a “yes” or “no” key with their right or left index finger respectively, and were required to respond as quickly as possible without compromising accuracy. Stimuli in the practice session were 10 objects (5 pictures, 5 words) that were not included in the main experiment. Following the practice session, participants were shown example photos of each object referent (i.e., shoebox, wheelie bin, pencil case) and were asked to report the average size of each referent.

The experiment consisted of four alternating study-test cycles (two relating to the wheelie bin referent change and two relating to the pencil case referent change) with each cycle lasting approximately 15 min. There was minimal break between cycles (typically just 10 s of seconds), to minimize the chance that participants forgot the instructions. During each study phase, 64 stimuli were shown two times resulting in 128 trials. 32 stimuli were presented as pictures (Within-format) and 32 were presented as words (Across-format). Words were presented in black on a white background with the same pixel dimensions as the pictures. Each set of 32 stimuli consisted of equal numbers of Congruent and Incongruent items. Apart from ensuring no immediate repetitions, the stimulus presentation order was randomized. Participants were always asked “is the object bigger than a shoebox?” at Study.

During each Test phase, the 64 stimuli from the Study phase (Within-format and Across-format) were randomly intermixed with 32 new stimuli (Novel). All items at Test were presented as pictures. Participants were either asked “is the object bigger than a wheelie bin?” or “is the object bigger than a pencil case?”. The order of task (i.e., type of reference object) was counterbalanced across participants in an ABBA/BAAB manner. When combined with the 3 stimulus sets, this meant 6 different counterbalancings (though owing to an experimenter error, only 5 of the 6 counterbalancings were used for the patients, with patients P1 and P3 having the same stimulus assignment). Throughout every trial during both Study and Test, the current task/reference was displayed at the top of the screen, so participants were unlikely to forget.

Each trial consisted of a 500 ms fixation cross followed by a stimulus that remained onscreen until the participant responded, followed by a blank screen for 200 ms. A response was required before the next trial started (i.e, the task was self-paced).

### 2.4 Analyses

Trials with RTs less than 400 ms, or two or more standard deviations above or below a participant's mean for a given task, were excluded from the RT analyses (also rendering the RT distributions more Gaussian). Given that there is some subjectivity in determining whether an object is bigger than a shoebox, wheelie bin or pencil case, errors were defined by a difference from the modal response for each object across participants in the Horner and Henson (2011) study.

Note that for Novel stimuli, “congruency” refers to whether the correct response for the “bigger”/“smaller” task would be the same or different for the study-task referent as for the test-task referent, even though participants never actually classified Novel items according to the study-task referent. Therefore the subtraction of Novel RTs from Repeated RTs for Congruent and Incongruent conditions separately means that priming effects were not confounded by item differences owing to how “close” in size each object was to the relevant referent (see Horner and Henson, 2011, for further details and analyses).

Error rates and RTs for correct trials at Test constituted the main dependent variables. Given the focus on S-R effects, RTs were further restricted to objects also given a correct judgment on both occurrences at Study. These variables were subjected to repeated-measures analyses of variance (ANOVAs). Only ANOVA effects that survived an alpha of 0.05 were reported, and unless stated otherwise, *t*-tests were two-tailed.

Two ANOVAs were performed: 1) a two-way ANOVA on Novel trials, with factors Group (Controls vs Patients) and Congruency (Congruent vs Incongruent), and 2) a three-way ANOVA on priming scores, with factors Group, Congruency and Format Match (Within vs Across format, ie whether items depicted as Pictures at Test had been seen as Pictures or Words respectively at Study). Because of potential differences in Congruent and Incongruent Novel RTs (as tested by the first ANOVA), as well as longer RTs for the Patient group than Control group, the two-way ANOVAs on RT priming scores were performed for three definitions of priming: subtractive, proportional and Z-scored. Subtractive priming is simply the difference in RTs for Repeated vs. Novel stimuli (Novel–Repeated); proportional priming is the difference in RTs for Repeated vs. Novel stimuli divided by Novel RTs ((Novel–Repeated)/Novel), calculated for each participant; Z-scored priming is obtained by taking each participant's condition means, subtracting their overall mean, and dividing by the standard deviation of their condition means (Faust et al., 1999). However, we focus on the proportional priming results because this was the measure used by Schnyer et al. (2006), and report subtractive and Z-scored priming in the Supplementary Material.

To ease comparison with Schnyer et al. (2006; Table 3), but in minor departures from Horner and Henson (2011; Table 1), we report standard errors rather than standard deviations, and express proportional priming in terms of %.

## 3 Results

### 3.1 Errors

The percentages of errors are shown in Table 2 (note that “errors” in this subjective size judgment are defined relative to the modal response; see Methods). The error rate in controls was very similar to those in Table 1 of Horner and Henson (2011), even though the present controls were considerably older. The error rate in controls was also comparable for congruent trials to that in Schnyer et al. (4.6%), but was numerically higher for incongruent trials than in Schnyer et al.’s reversed condition (7.2%). The latter likely reflects the additional interference from reversing S-C bindings in the present paradigm but not in the Schnyer et al. paradigm. The error rate for patients in the present study was very similar to that in controls, suggesting that the patients could perform the task at a similar level. This contrasts with the patients in the Schnyer et al. study, who made more errors (11.5% and 10.2% for maintained versus reversed tasks respectively) than their controls for both tasks.

**Table 2:** Mean percentage errors and reaction times (RTs), with standard errors in parentheses, for Within-format, Across-format and Novel conditions of Experiment 1, plus error priming, (subtractive) RT priming and proportional RT priming (% Priming) as a function of Congruency (Con, Inc). Note that for Novel stimuli, “congruency” refers to whether the correct response for the “bigger”/“smaller” task would be the same or different for the study-task referent as for the test-task referent, even though participants never actually classified Novel items according to the study-task referent.

Condition/Congruency	Within-format (Picture-Picture)		Across-format (Word-Picture)		Novel	
	Con	Inc	Con	Inc	Con	Inc
Controls (N = 24)						
*% Errors*	5.67 (0.80)	13.9 (1.44)	5.13 (0.92)	14.6 (1.56)	5.04 (0.76)	14.4 (1.36)
*Error Priming*	−0.63 (0.70)	0.5 (1.07)	−0.08 (0.71)	−0.17 (0.98)		
						
Patients (N = 6)						
*% Errors*	4.33 (0.88)	17.3 (3.16)	4.67 (0.95)	14.3 (2.75)	4.17 (1.38)	15.8 (2.73)
*Error Priming*	−0.17 (1.78)	−1.50 (3.02)	−0.50 (1.61)	1.50 (1.61)		
						
Controls (N = 24)						
*RTs*	814 (29)	1034 (48)	882 (35)	1113 (56)	916 (38)	1044 (46)
*RT Priming*	103 (18)	10 (23)	34 (15)	−69 (25)		
*RT % Priming*	10.4 (1.47)	0.62 (2.09)	3.23 (1.45)	−6.38 (2.12)		
						
Patients (N = 6)						
*RTs*	1433 (480)	2076 (669)	1636 (490)	2388 (923)	1725 (642)	1889 (564)
*RT Priming*	292 (168)	−187 (110)	88 (160)	−499 (361)		
*RT % Priming*	12.8 (4.54)	−7.59 (2.71)	−2.10 (4.76)	−15.5 (7.95)		

The 2 × 2 ANOVA on error rates in Novel conditions showed only a significant main effect of Congruency, F(1,28) = 41.7, p < 0.001, which reflected more errors in the Incongruent than Congruent condition. This was expected, since these items tend to be closer to the referents and hence more ambiguous, and illustrates the importance of having separate Novel baselines with which to measure priming. There was no significant effect of Group (Controls vs Patients), nor interaction between Group and Congruency, F(1,28)’s < 1.

The 2 × 2 × 2 ANOVA on subtractive priming in error rates showed no significant effects or interactions between Format Match, Congruency and Group, F(1,28)’s < 2.88, p's > 0.10. These results suggest that the RT priming effects below are unlikely to reflect a speed-accuracy trade-off.

The results of the same analyses repeated with sex as a covariate were very similar (see Supplementary Material).

### 3.2. Reaction times

**Fig. 2:**
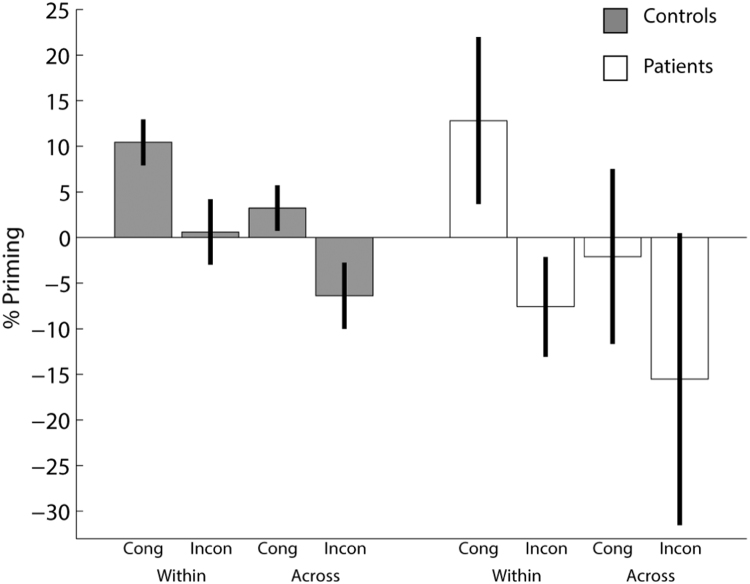
Proportional Priming for each condition and group. Cong = Congruent; Incon = Incongruent. Error bars are one-tailed 95% confidence intervals, one-tailed, given the prior patterns in Horner and Henson (2011). For individual patient data, see Fig. 3 and Supplementary Material.

In additional to the errors described above, we excluded another 12% of trials in the Control group and 8% in the Patient group with outlying RTs in Test trials or inconsistent responses across Study trials (see Methods). The average number of remaining (correct) trials per condition was 50 (min = 21, max = 62) for controls and 53 (min = 35, max = 63) for patients. As expected, the present controls were slower (Table 2) than their younger counterparts in Table 1 of Horner and Henson (2011), which might explain why their subtractive priming scores were slightly larger, though their proportional priming scores were more comparable. Patients were slower still (than the present controls), though there was large variability across patients (see Supplementary Table 1 for scores for each patient). Nonetheless, proportional priming was similar in size to the controls, if slightly more extreme (Fig. 2).

The 2×2 ANOVA on Novel conditions showed a significant main effect of Congruency, F(1,28) = 21.2, p < 0.001, which reflected longer RTs in the Incongruent than Congruent condition, as expected (and paralleling the increased error rate reported above). There was also a significant main effect of Group, F(1,28) = 7.81, p < 0.01, which reflected longer RTs in patients than controls. There was no evidence for an interaction between Congruency and Group, F(1,28) < 1. The slower RTs to Novel items for both Incongruent relative to Congruent conditions, and for patients relative to controls, reinforces the importance of measuring priming by proportional means below.

#### 3.2.1. Proportional priming

The 2 × 2 × 2 ANOVA on proportional priming showed a significant main effect of Congruency, F(1,28) = 23.2, p<; .001, with positive priming (response speeding) for Congruent conditions and negative priming (response slowing) for Incongruent conditions, together with a significant main effect of Format Match, F(1,28) = 30.5, p < 0.001, with more positive priming within formats rather than across formats, as expected. Unlike Horner and Henson (2011), any interaction between Congruency and Format Match did not reach significance, F(1,28) < 1. There was a significant main effect of Group, F(1,28) = 4.38, p < 0.05, with patients showing more negative priming scores on average (see Fig. 2). Interestingly, there was no evidence for an interaction between Group and Congruency, F(1,28) = 1.55, p = 0.224, nor for an interaction between Group and Format Match, F(1,28) = 1.43, p = 0.241, nor for the three-way interaction, F(1,28) = 1.24, p = 0.274. These results suggest that patients exhibit similar proportional priming effects as controls, i.e, were equally sensitive to S-R effects (see also Bayes Factor analyses below). Indeed, if anything, the numerical size of the mean congruency effect on priming (averaged across Format Match) was larger, rather than smaller, in the Patient group (M = 16.9%) than Control group (M = 9.72%).

The significance of proportional priming effects in each of the four conditions separately is illustrated in Fig. 2 (error bars are 95% confidence intervals). Like the young controls in Horner and Henson (2011), the present older controls showed significant positive priming in the two Congruent conditions, and significant negative priming in the Incongruent Across-format condition. The patients showed significant positive priming in the Congruent Within-format condition, but not the Congruent Across-format condition. The patients also showed negative priming, though this only reached significance in the Within-format Incongruent condition (p = 0.054 for the Across-format Incongruent condition). Thus not only was there no evidence from the previous ANOVA that the pattern of priming differed across groups, but there were cases of both positive and negative priming that were significant even when the patient group was considered on its own.

The same pattern of significant effects was seen when using either Subtractive Priming or Z-scored Priming (see Methods), or when sex was covaried out, as shown in Supplementary Material. One patient (P3) showed particularly long RTs (Supplementary Table 1), so to further check that results were not driven by this patient, we repeated the ANOVA on proportional priming without P3. There was still no two-way interaction between Congruency and Group, and the congruency effect was still numerically larger in the Patient group (see Supplementary Material). Finally, to more closely match the Schnyer et al. paradigm, we repeated the ANOVA on our Within-Format conditions only, but again found no evidence of a smaller Congruency effect in patients than controls (see Supplementary Material).

We conducted various additional analyses to confirm the significance of the proportional priming results.

#### 3.2.2. Nonparametric tests

Given that the patient group only had 6 members, while the control group had 24, it is difficult to assess the homogeneity of variance assumed by the above (parameteric) ANOVAs. We therefore performed non-parametric, Wilcoxon ranksum tests on proportional priming scores, separately for each of the within-group effects of 1) Congruency, 2) Format Match and 3) Congruency-by-Format-Match interaction. When combining both groups, there were significant main effects of Congruency, ranksum = 427, p < 0.001, and Format Match, ranksum = 437, p < 0.001, but no interaction between these two factors, ranksum = 281, p = 0.32. When comparing the two groups however, there was no evidence for any interaction between Group and any of these three effects, ranksums < 346, p's > 0.19.

#### 3.2.3. Individual patient tests

In case of important differences between the six patients, we also compared the congruency effect in each patient separately against the control group, using a T-test with pooled variance (sometimes called Crawford's Test). When averaging across Format Match, only one patient was significantly different from controls (Supplementary Table 2), which was P3, who was the patient who showed longer RTs in total (see ANOVA above with P3 excluded). Importantly, this patient actually showed a greater effect of congruency on priming than the controls (i.e., not the smaller effect reported by Schnyer et al.). The same pattern of significant results obtained when analysing the Within-format condition only, to closer match the Schnyer et al. study, with only P3 showing a significantly greater congruency effect than controls (Supplementary Table 2). Given that the precise way to adjust priming scores by overall RTs is a matter of debate (Faust et al., 1999), we do not pursue this further, but simply note that individual analyses provide no support for the hypothesis that hippocampal lesions reduce the influence of S-R bindings, and group analyses show that our results are not overly influenced by the patient with exceptionally long RTs.

#### 3.2.4. Bayes factor

The lack of a significant two-way interaction between Congruency and Group suggests that the congruency effect, as an index of S-R bindings, is comparable in our patients compared to our controls. To provide more evidence for this conclusion, rather than relying on failure to reject the null hypothesis that the two groups differ, we calculated the Bayes Factor for the likelihood of patients showing the same size congruency effect as controls (alternate hypothesis), relative to the likelihood of patients showing no congruency effect (null hypothesis; Dienes, 2011). Given the congruency effect for proportional priming (averaged across format) had a mean of 19.4% and standard deviation of 21.4% in controls, and a mean of 33.8% and standard deviation of 38.7% in patients, the Bayes Factor was 5.09, which provides “substantial” (Jeffreys, 1961) evidence for the hypothesis that controls and patients show the same size congruency effect.

#### 3.2.5. Comparison with Schnyer et al. (2006)

**Fig. 3:**
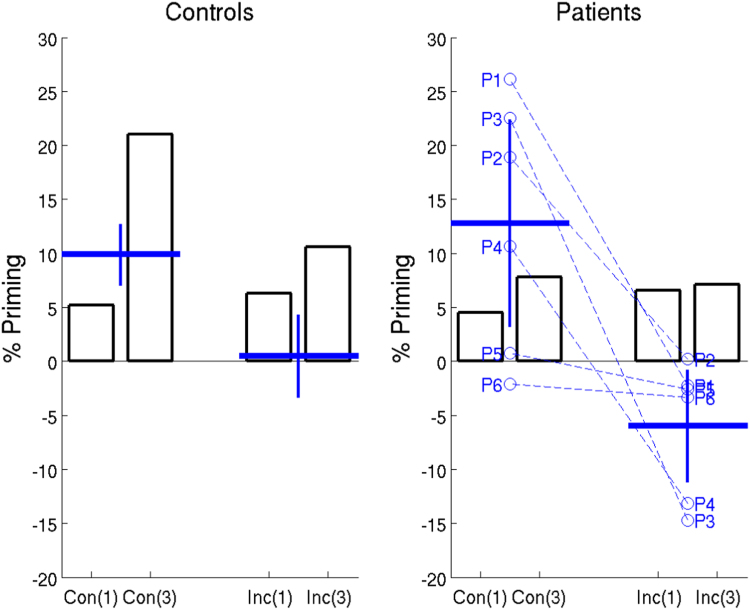
Comparison of proportional priming effects in current study with that of Schnyer et al. (2006; Experiment 2) for Congruent (Con) and Incongruent (Inc) trials. The open bars are data replotted from Block 1 of Schnyer et al., for their Low Primed (1 study trial, e.g, “Con(1)”) and High Primed (3 study trials, e.g, “Con(3)”) conditions (N = 12 controls and N = 9 patients; no SD data provided); the blue horizontal lines and error bars are means and 95% one-tailed confidence intervals from the Within Format condition of the present study (N = 24 controls and N = 6 patients), after adjusting for sex. Data from the six patients (P1-P6) from the present study are also plotted separately. (For interpretation of the references to color in this figure legend, the reader is referred to the web version of this article).

For direct comparison with Experiment 2 of Schnyer et al. (2006), we re-plotted the proportional priming scores from their study together with those from the present study. We took data from their Block 1, and from our Within-Format conditions, since these are the most comparable conditions. The results are shown in Fig. 3. The main difference for the controls is that the Incongruent condition abolished priming in the present study, but not the Schnyer et al. study, consistent with our claim that the present design reverses multiple levels of response representation, including Stimulus-Classification bindings, which are not reversed in the Inverted condition of Schnyer et al. More importantly, reversing multiple levels of response representation also reduced priming in all six of the present patients, unlike the patients in the Schnyer et al. study, who showed no effect of decision and action reversal.

## 4 Discussion

We fully expected, based on Schnyer et al. (2006), that individuals with hippocampal damage would show reduced evidence of stimulus-response (S-R) bindings. S-R bindings were indexed by the effect of congruency on RT priming, which reflects the difference in priming when the response is reversed between initial and repeated stimulus presentations, relative to when it is maintained. Our expectations were not confirmed however, in that we found that the evidence favoured the null hypothesis of equivalently-sized congruency effects in patients and controls, and the patients clearly showed significant congruency effects, no matter how we measured them. This finding is surprising, because according to many theories, the hippocampus would seem to be a critical brain structure for rapid learning (from just 1–2 presentations) of novel and arbitrary S-R bindings.

We start by considering possible reasons for the discrepancy between the Schnyer et al. (2006) study and the present study, before discussing other findings and the broader implications of our study.

### 4.1 Possible differences from Schnyer et al. (2006)

One possible difference between the present study and that of Schnyer et al. (2006) concerns the type of patients. For example, the patients tested by Schnyer et al. (2006) may have had more severe or extensive lesions of hippocampus, such that a threshold for observing reduced S-R bindings was exceeded. A direct comparison is not possible because Schnyer et al. (2006) were unable to report details of the extent of hippocampal damage. However it is noteworthy that the average, bilateral gray-matter volume loss in hippocampus was 40% in the present cases, which is comparable to other studies of acquired hippocampal damage in human adults. Moreover, all six of the present cases showed marked impairment on at least one standardised neuropsychological test of memory (though unfortunately, these were different tests from those used by Schnyer et al., so again, direct comparison is difficult). Thus while we cannot refute the possibility that the patients in the Schnyer et al. study had greater hippocampal damage and more severe amnesia, the present hippocampal lesions were sufficiently severe to impair episodic memory, and so it remains interesting that there was no corresponding impairment detected on S-R binding.

Another possibility is that the extent of damage outside the hippocampus was greater in Schnyer et al.'s patients; for example, spreading into surrounding perirhinal cortex. Perirhinal cortex may play a role in binding stimuli and responses, given evidence that this region supports not only representations of complex visual objects, but also the encoding of object-context associations (Watson et al., 2012). If the patients in Schnyer et al.’s study had greater perirhinal damage, then they would show stronger evidence of impairment of S-R bindings. One problem for this perirhinal account however is the finding reported by Wang et al. (2010). These authors compared priming for patients with hippocampal lesions against priming for patients with larger MTL lesions, using the same object-size judgment task as used here and by Schnyer et al. (2006) (though on words at both study and test). Wang et al. did not manipulate response congruency, so S-R bindings were likely to contribute to their priming measure. While their hippocampal group showed priming (consistent with the priming found in the congruent conditions of the present study and of Schnyer et al.), their MTL group did not show significant priming, which Wang et al. attributed to impaired conceptual processing in perirhinal cortex (based on additional fMRI data). If the Wang et al. results hold, then the fact that Schnyer et al.'s patients did show significant priming suggests that they did not have significant perirhinal damage.

Another possibility is that the key region of damage in the Schnyer et al. (2006) patients but not present patients is entorhinal cortex. This would be consistent with lesion work in non-human primates that has investigated rapid learning of arbitrary visuo-motor mappings (visuomotor learning, VML), which resemble S-R bindings. Although initial work using aspiration lesions of the hippocampus plus underlying cortex, including caudal entorhinal cortex, indicated that these regions were critical (Murray and Wise, 1996, Murray and Mishkin, 1998), subsequent research using more precise, reversible inactivation via intracerebral infusion of a GABA agonist found that hippocampus was not essential for VML; rather entorhinal cortex was key (Yang et al., 2014, Murray and Mishkin, 1998). Only 2 of the 6 patients in the present study (P4 and P6) had significant entorhinal volume loss detectable from MRI (compared to all 6 for hippocampal volume loss, Henson et al., 2016). When testing individually, there was no evidence that P4 or P6 showed reduced congruency effects, though one should be wary of null results in single cases. Note that, even if the presence or absence of entorhinal damage does reconcile results across the two studies, the present study would still be correct in concluding that hippocampus proper is not necessary for S-R bindings.

Other NHP studies have shown that the fornix is also critical for VML (Brasted et al., 2005), yet diffusion-weighted MRI showed that the present group of 6 patients also had significant white matter abnormalities of the fornix, in addition to their hippocampal damage (Henson et al., 2016). Thus the relationship between the neural correlates of S-R bindings in humans and neural correlates of VML in non-human primates requires further investigation.

A final anatomical possibility is that the patients in Schnyer et al.’s study (but not those in the present study) also had damage to basal ganglia circuits that support many types of motor/habit learning (e.g., Gabrieli, 1998; Poldrack et al., 2001). However, motor (procedural) learning is conventionally thought to be more gradual (i.e., incremental over many trials) than the type of S-R learning occurring here; a point we return to later.

A second dimension along which the two studies differ concerns the paradigm. We reversed response contingencies by changing the referent of the size judgment task, rather than reversing the direction of the size judgment. As explained in the Introduction, the reason for this change was that we have previously shown (Horner and Henson, 2009, Horner and Henson, 2011) that a referent change additionally invalidates any bindings between the stimulus and the “classification” response (i.e., the label “bigger” or “smaller”), so the present referent change arguably induces a more comprehensive disruption of the use of S-R bindings. This could explain why priming was completely abolished in the present Incongruent (Within Format) condition, whereas it remained significant (though reduced) in the corresponding (Inverted) condition of the Schnyer et al. (2006) study. Indeed, the use of Stimulus-Classification (S-C) bindings might be the cause of this residual priming in Schnyer et al.’s data, rather than a residual conceptual/perceptual processing component. Nonetheless, it remains possible that reversing the task instructions has a qualitatively different effect (e.g, in terms of task switching, Henson et al., 2016) from changing the referent; more specific hypotheses would be needed to test this.

Another difference between the two paradigms concerns the number of study presentations (before the task or referent is changed). Schnyer et al. used both a single presentation (their Low Prime condition) and three presentations (their High Prime condition), whereas we used two presentations. One possibility is that two study repetitions were sufficient for non-hippocampal S-R learning (e.g., via basal ganglia). However, this is contrary to the effect that the number of presentations had on Schnyer et al.'s results: Although they did not analyse their High and Low conditions separately, the numerical pattern for their single presentation condition actually showed little effect of task reversal in Block 1 for either patients or controls; it was only after three presentations that Schnyer et al. observed a greater task reversal cost in controls than patients. Thus another way to reconcile the two studies is to propose that the difference between patients and controls only emerges after three or more presentations. In other words, three presentations may be necessary for controls to form an explicit representation of an S-R contingency in the hippocampus. However, this is the opposite to 1) conventional views that the hippocampus supports one-shot learning, and that other (eg basal ganglia) systems support more gradual, procedural learning (Gabrieli, 1998), and 2) other studies in healthy controls, which found only quantitative rather than qualitative effects of a small number of repetitions on S-R learning (e.g., Horner and Henson, 2009; Dennis and Perfect, 2012).

A final possibility is that the discrepant results across the two studies reflect statistical artefacts. It is possible that our failure to find an interaction between controls vs patients and congruent vs incongruent conditions was a type II error (given that we only tested 6 patients, whereas Schnyer et al. tested 9). However, our Bayes Factor analysis favours the null hypothesis of no interaction. Moreover, the three-way interaction between Group, Cue (Congruency) and Condition (Number of Repetitions) was not actually significant in Schnyer et al.'s study either; their main claim was based on the fact that their Control group showed a significant Cue-by-Condition interaction, but their Patient group did not. Thus it is also possible that Schnyer et al.'s failure to find evidence for S-R bindings in the patients was a type II error (whereas here, all 6 of the patients showed evidence of S-R bindings). Ultimately, a direct replication of Schnyer et al.'s paradigm is necessary to resolve the apparent discrepancies with the current paradigm.

### 4.2 Further findings

We introduced the within- versus across-format manipulation to test whether hippocampal lesions would affect the ability to generalise S-R bindings across stimulus codes, as well as across response codes. Unlike our previous study in young controls (Horner and Henson, 2011), we failed to find evidence of a greater effect of congruency in the within- than across-format conditions (i.e, no significant interaction between Congruency and Format Match on proportional priming). Thus there was no evidence in the present study of format-specific stimulus codes. Nonetheless, the congruency effect was significant in the Across-Format conditions alone, F(1,28) = 12.5, p < 0.001 (with no evidence of an interaction with Group, F(1,28) < 1), indicating that S-R bindings generalised across the format of word versus picture. One might note that priming in the Congruent Across-Format condition was not significant for the patients, as it was for controls (see Fig. 2). However, this cannot be used to conclude that hippocampal lesions disrupt abstract stimulus codes, because there was no evidence that proportional priming in the Across-Format condition was smaller in patients than controls, T(28) = 1.07, p = 0.29, and the lack of significant priming in the patient group alone could simply reflect low power. Moreover, S-R bindings are indexed by the Congruency effect (i.e, Congruent vs Incongruent); direct comparisons between groups for single conditions (e.g, the Congruent Across-Format condition), even if they were reliable, could reflect group differences in mechanisms other than S-R bindings, such as the facilitation of semantic component processes.

While we think it is important that our changing referent paradigm reverses all three levels of response representation currently known (Horner and Henson, 2009), e.g. in order to address the possibility that the patients in the Schnyer et al. (2006) study were still using stimulus-classification (S-C) bindings, we cannot reject the possibility that the congruency effects in the changing referent paradigm are driven by one type of response code alone (rather than all three). For example, priming in this paradigm might be dominated by stimulus-action (S-A) bindings. This is important because our findings would then also be consistent with the possibility that hippocampal lesions do not affect S-A bindings (producing congruency effects comparable to controls in the present paradigm), but do affect stimulus-decision (S-D) and/or S-C bindings (that are just not detectable in the present paradigm). Indeed, in this case, it is further possible that the Schnyer et al. (2006) paradigm is, for some reason, dominated instead by S-D bindings, such that an effect of hippocampal lesions is seen in that paradigm. While we have no evidence for such a difference between paradigms when we have used both (see Horner and Henson, 2009), testing these possibilities would require further experiments in patients that dissect the different levels of response codes in S-R bindings, like the experiments in Horner and Henson (2009).

Another important consideration concerns the possible role of Stimulus-Task (S-T) bindings (Henson et al., 2014). While S-T bindings are normally conceived at the abstract level of task (e.g, size judgments versus animacy judgments), it is possible that S-T bindings are formed that are specific to the referent (e.g, associating a picture of a monkey with a “Bigger_Than_Shoebox” task label, rather than just “Bigger” classification label). While such S-T bindings could not cause the present congruency effect, because the referent changes in both Congruent and Incongruent conditions (see Fig. 1), they could contribute to the congruency effect reported (in Controls) by Schyner et al. (2006), where S-T bindings would change in the Incongruent but not Congruent condition. Thus if hippocampal lesions affect such specific S-T bindings, this could explain why the patients in Schnyer et al. did not show a congruency effect. If these lesions did not affect other types of S-R bindings (e.g, S-D or S-A bindings), then this could further explain why patients did show a congruency effect in the present study, though it would remain unclear why the integrity of such other S-R bindings did not also cause a congruency effect in the Schnyer et al. study. We hope that future researchers will investigate the importance of the various differences that we listed above between our study and Schnyer et al.'s, including running direct replications of both studies.

### 4.3 Implications

We think that evidence for S-R bindings, like that presented here, implies an impressive ability of the human brain to rapidly learn a large number of S-R mappings. Of course, we do not know that an S-R binding was formed or retrieved for every trial in the experiment – it is possible that only a small fraction of trials in which S-R retrieval occurred was sufficient to cause the average RT priming effects – but at the other extreme, the implication is that the brain can store hundreds of unique mappings. So if the hippocampus is not the brain structure that supports this impressive feat, which brain region is?

As mentioned above, one possibility is the surrounding entorhinal or perirhinal cortex, consistent with the animal lesions studies using the VML task, while another possibility is basal ganglia structures, which have previously been associated with procedural learning (though normally associated with more gradual learning, they may be able to learn enough from 1 to 2 stimulus-response pairings to produce a detectable effect on RTs). A third possibility, not considered above, is that S-R bindings are mediated by prefrontal regions. This would be consistent with human fMRI and M/EEG studies of S-R retrieval, which implicate ventral prefrontal regions in particular (e.g., Horner and Henson, 2008; 2012; Race et al., 2009; Wig et al., 2009). It would also be consistent with evidence that ventral (and orbital) prefrontal lesions in animals impair VML (Bussey et al., 2001). It would therefore be interesting to run the present paradigm in human patients with prefrontal lesions, where significantly smaller congruency effects would be predicted.

Theoretically, S-R bindings may not conform to the type of flexible associations attributed to hippocampus (Cohen et al., 1997); associations that can be voluntarily retrieved and inter-related (e.g, to make transitive inferences across associations). While S-R bindings are clearly complex, encoding several types of response representations (Horner and Henson, 2009) and relatively abstract stimulus representations (as shown by the congruency effect in the present Across Format conditions), they may be relatively inflexible, in the sense of being retrieved automatically and independently. A related possibility is that S-R bindings are not represented explicitly, in the sense of participants being aware of them, which is why hippocampus is not involved. While we cannot rule out the possibility that both controls and patients had episodic memories for some trials (those in which S-R bindings were formed), the fact that S-R bindings co-occur with impairments on standard tests of episodic (explicit) memory in the present patients suggests that S-R bindings are more likely to be implicit (see also Giesen and Rothermund, 2015). In short, the inflexible, involuntary and/or implicit nature of S-R bindings may indicate a non-hippocampal locus; but nonetheless a locus that allows rapid encoding of multiple arbitrary mappings. This reinforces the point that it is theoretically important to understand not only what individuals with amnesia cannot to, but also what they can do (Clark and Maguire, 2016).

## Footnotes

1 Wheelie bin is a common term in the United Kingdom that refers to a large trash can (with wheels), which has a standard size (approx 1 m × 0.6 m × 0.7 m; 240 l) that would be well-known by our participants.

## References

[bib1] Allenmark F., Moutsopoulou K., Waszak F. (2015). A new look on S-R associations: How S and R link. Acta Psychol. (Amst).

[bib2] Brasted P.J., Bussey T.J., Murray E.A., Wise S.P. (2005). Conditional motor learning in the nonspatial domain: effects of errorless learning and the contribution of the fornix to one-trial learning. Behav. Neurosci..

[bib3] Bussey T.J., Wise S.P., Murray E.A. (2001). The role of ventral and orbital prefrontal cortex in conditional visuomotor learning and strategy use in rhesus monkeys (Macaca mulatta). Behav. Neurosci..

[bib4] Cave C.B., Squire L.R. (1992). Intact and long-lasting repetition priming in amnesia. J. Exp. Psychol. Learn. Mem. Cogn..

[bib5] Clark I.A., Maguire E.A. (2016). Remembering preservation in hippocampal amnesia. Annu. Rev. Psychol..

[bib6] Cohen N.J., Eichenbaum H.B. (1994). Memory, Amnesia and the Hippocampal System.

[bib7] Cohen, N.J., Poldrack, R.A., Eichenbaum, H. Memory for Items and Memory for Relations in the Procedural/Declarative Memory Framework Memory, 5, 1997, pp. 131–178. 10.1080/741941149 9156097

[bib8] Denkinger B., Koutstaal W. (2009). Perceive-decide-act, perceive-decide-act: how abstract is repetition-related decision learning?. J. Exp. Psychol. Learn. Mem. Cogn..

[bib9] Dennis I., Perfect T.J. (2012). Do stimulus–action associations contribute to repetition priming?. J. Exp. Psychol. Learn. Mem. Cogn..

[bib10] Dienes Z. (2011). Bayesian versus orthodox statistics: which side are you on?. Perspect. Psychol. Sci..

[bib11] Dobbins I.G., Schnyer D.M., Verfaellie M., Schacter D.L. (2004). Cortical activity reductions during repetition priming can result from rapid response learning. Nature.

[bib12] Faust M.E., Balota D.A., Spieler D.H., Ferraro F.R. (1999). Individual differences in information-processing rate and amount: implications for group differences in response latency. Psychol. Bull..

[bib13] Gabrieli J.D. (1998). Cognitive neuroscience of human memory. Annu. Rev. Psychol..

[bib14] Giesen C., Rothermund K. (2015). Adapting to stimulus–response contingencies without noticing them. J. Exp. Psychol. Hum. Percept. Perform..

[bib15] Giovanello K.S., Schnyer D.M., Verfaellie M. (2004). A critical role of the anterior hippocampus in relational memory: Evidence from an fMRI study comparing associative and item recognition. Hippocampus.

[bib16] Henson R.N., Eckstein D., Waszak F., Frings C., Horner A.J. (2014). Stimulus-response bindings in priming. Trends Cogn. Sci..

[bib17] Henson R.N., Greve A., Cooper E., Gregori M., Simons J.S., Geerligs L. (2016). The effects of hippocampal lesions on MRI measures of structural and functional connectivity. Hippocampus.

[bib18] Horner A.J., Henson R.N. (2008). Priming, response learning and repetition suppression. Neuropsychologia.

[bib19] Horner A.J., Henson R.N. (2009). Bindings between stimuli and multiple response codes dominate long-lag repetition priming in speeded classification tasks. J. Exp. Psychol. Learn. Mem. Cogn..

[bib20] Horner A.J., Henson R.N. (2011). Stimulus-response bindings code both abstract and specific representations of stimuli: evidence from a classification priming design that reverses multiple levels of response representation. Mem. Cogn..

[bib21] Horner A.J., Henson R.N. (2012). Incongruentabstract stimulus-response bindings result in response interference: fMRI andEEG evidence from visual object classification priming. J. Cogn. Neurosci..

[bib22] Koutstaal W., Wagner A.D., Rotte M., Maril A., Buckner R.L., Schacter D.L. (2001). Perceptual specificity in visual object priming: functional magnetic resonance imaging evidence for a laterality difference in fusiform cortex. Neuropsychologia.

[bib23] Moscovitch, M. Memory and working with memory: evaluation of a component process model and comparisons with other models. In: Memory Systems, 1994, p. 269–310.

[bib24] Murray, EA, Mishkin, M. Object Recognition and Location Memory in Monkeys with Excitotoxic Lesions of the Amygdala and Hippocampus, 18, 1998, pp. 6568–6582. 10.1523/JNEUROSCI.18-16-06568.1998 PMC6793180 9698344

[bib25] Murray E., Wise S. (1996). Role of the hippocampus plus subjacent cortex but not amygdala in visuomotor conditional learning in rhesus monkeys. Behav. Neurosci..

[bib26] Poldrack R.A., Clark J., Paré-Blagoev E.J., Shohamy D., Creso Moyano J., Myers C. (2001). Interactive memory systems in the human brain. Nature.

[bib27] Race E.A., Shanker S., Wagner A.D. (2009). Neural priming in human frontal cortex: multiple forms of learning reduce demands on the prefrontal executive system. J. Cogn. Neurosci..

[bib28] Schacter D.L., Buckner R.L. (1998). Priming and the brain. Neuron.

[bib29] Schacter D.L., Chiu C.Y., Ochsner K.N. (1993). Implicit memory: a selective review. Annu. Rev. Neurosci..

[bib30] Schacter D.L., Tulving E. (1994). What are the memory systems of 1994?. Mem. Syst..

[bib31] Schnyer D.M., Dobbins I.G., Nicholls L., Davis S., Verfaellie M., Schacter D.L. (2007). Item to decision mapping in rapid response learning. Mem. Cogn..

[bib32] Schnyer D.M., Dobbins I.G., Nicholls L., Schacter D.L., Verfaellie M. (2006). Rapid response learning in amnesia: delineating associative learning components in repetition priming. Neuropsychologia.

[bib33] Simons J.S., Koutstaal W., Prince S., Wagner A.D., Schacter D.L. (2003). Neural mechanisms of visual object priming: evidence for perceptual and semantic distinctions in fusiform cortex. Neuroimage.

[bib34] Squire L.R. (1992). Memory and the hippocampus: a synthesis from findings with rats, monkeys, and humans. Psychol. Rev..

[bib35] Wang W.C., Lazzara M.M., Ranganath C., Knight R.T., Yonelinas A.P. (2010). The medial temporal lobe supports conceptual implicit memory. Neuron.

[bib36] Watson H.C., Wilding E.L., Graham K.S. (2012). A role for perirhinal cortex in memory for novel object-context associations. J. Neurosci..

[bib37] Wig G.S., Buckner R.L., Schacter D.L. (2009). Repetition priming influences distinct brain systems: evidence from task-evoked data and resting-state correlations. J. Neurophysiol..

[bib38] Yang T., Bavley R.L., Fomalont K., Blomstrom K.J., Mitz A.R., Turchi J. (2014). Contributions of the hippocampus and entorhinal cortex to rapid visuomotor learning in rhesus monkeys. Hippocampus.

