# Supplementary material for: No effect of hippocampal lesions on stimulus-response bindings
Source: Neuropsychologia. 2017 Aug;103:106–14. doi: 10.1016/j.neuropsychologia.2017.07.024 (PMC5726084; doi:10.1016/j.neuropsychologia.2017.07.024)
Supplement: Supplementary file 1 — Supplementary material [file mmc1.doc]

# Supplementary Material

## Supplementary Table 1: Each patient’s error and RT data separately

| Condition / | Within-format  (Picture-Picture) | | Across-format  (Word-Picture) | | Novel | |
| --- | --- | --- | --- | --- | --- | --- |
| Congruency | Con | Incon | Con | Incon | Con | Incon |
|  |  |  |  |  |  |  |
| *% Errors* |  |  |  |  |  |  |
| *P1* | 2 | 17 | 3 | 11 | 6 | 8 |
| *P2* | 3 | 13 | 6 | 6 | 0 | 9 |
| *P3* | 8 | 30 | 3 | 19 | 2 | 20 |
| *P4* | 5 | 14 | 6 | 14 | 2 | 22 |
| *P5* | 5 | 8 | 8 | 11 | 9 | 13 |
| *P6* | 3 | 22 | 2 | 25 | 6 | 23 |
|  |  |  |  |  |  |  |
| *RTs (ms)* |  |  |  |  |  |  |
| *P1* | 783 | 1155 | 974 | 1000 | 1042 | 1086 |
| *P2* | 1146 | 1834 | 1549 | 2127 | 1410 | 1790 |
| *P3* | 3810 | 5329 | 4032 | 6893 | 4906 | 4623 |
| *P4* | 1066 | 1920 | 1313 | 2017 | 1204 | 1672 |
| *P5* | 1029 | 1241 | 1114 | 1313 | 1009 | 1259 |
| *P6* | 765 | 977 | 836 | 978 | 777 | 904 |

## Supplementary Table 2: Each patient’s priming separately

| Condition / | Within-format  (Picture-Picture) | | Across-format  (Word-Picture) | |
| --- | --- | --- | --- | --- |
| Congruency | Con | Incon | Con | Incon |
|  |  |  |  |  |
| *Proportional Priming of RTs (%)* | | | | |
| *P1* | 24.8 | -6.35 | 6.53 | 7.92 |
| *P2* | 18.7 | -2.46 | -9.86 | -18.8 |
| *P3* | 22.3 | -15.3 | 17.8 | -49.1 |
| *P4* | 11.5 | -14.8 | -9.05 | -20.6 |
| *P5* | -1.98 | 1.43 | -10.4 | -4.29 |
| *P6* | 1.54 | -8.08 | -7.59 | -8.19 |
| *Congruency Effect on Priming for Controls - Patient (df=23)* | | | | |
|  | Collapse Format | | Within Format | |
|  | T-value | P-value | T-value | P-value |
| *P1* | -0.47 | .64 | -1.79 | .087 |
| *P2* | -0.49 | .63 | -0.95 | .35 |
| *P3* | -3.89* | .0007 | -2.32* | .029 |
| *P4* | -0.84 | .41 | -1.38 | .18 |
| *P5* | 1.32 | .20 | 1.11 | .28 |
| *P6* | 0.42 | .68 | 0.02 | .99 |

## Subtractive Priming

We also analysed a subtractive rather than proportional measure of priming. The same 2x2x2 ANOVA on subtractive priming showed a significant main effect of Congruency, F(1,28)=5.84, p<.05, together with a significant main effect of Format Match, F(1,28)=13.2, p<.001, and highly significant main effect of Group, F(1,28)=9.98, p<.005. Again, any interaction between Congruency and Format Match did not reach significance; nor did the three-way interaction between Congruency, Format Match and Group, F(1,28)’s<1. The two-way interactions between Group and Congruency, F(1,28)=5.15, p<.05, and between Group and Format Match, F(1,28)=5.87, p<.05, did reach significance, unlike the results for proportional priming. Importantly though, these interactions reflected a larger effect of Congruency and of Format Match on subtractive priming for Patients than for Controls. In other words, subtractive priming suggested that Patients were actually *more* sensitive to S-R effects than were Controls (if slower RTs overall do not matter).

## Z-scored Priming

We also analysed a Z-scored measure of priming, which was recommended by Faus*t et a*l. (1999) in preference to proportional scoring. The 2x2x2 ANOVA on subtractive priming after Z-scoring showed a significant main effect of Congruency, F(1,28)=17.7, p<.001, together with a significant main effect of Format Match, F(1,28)=42.0, p<.001, but no significant main effect of Group, F(1,28)=1.95, p=.174. No other interaction reached significance, F(1,28)’s<1.2, p’s>.28. For pattern of significant priming effects, see Supplementary Figure 1.


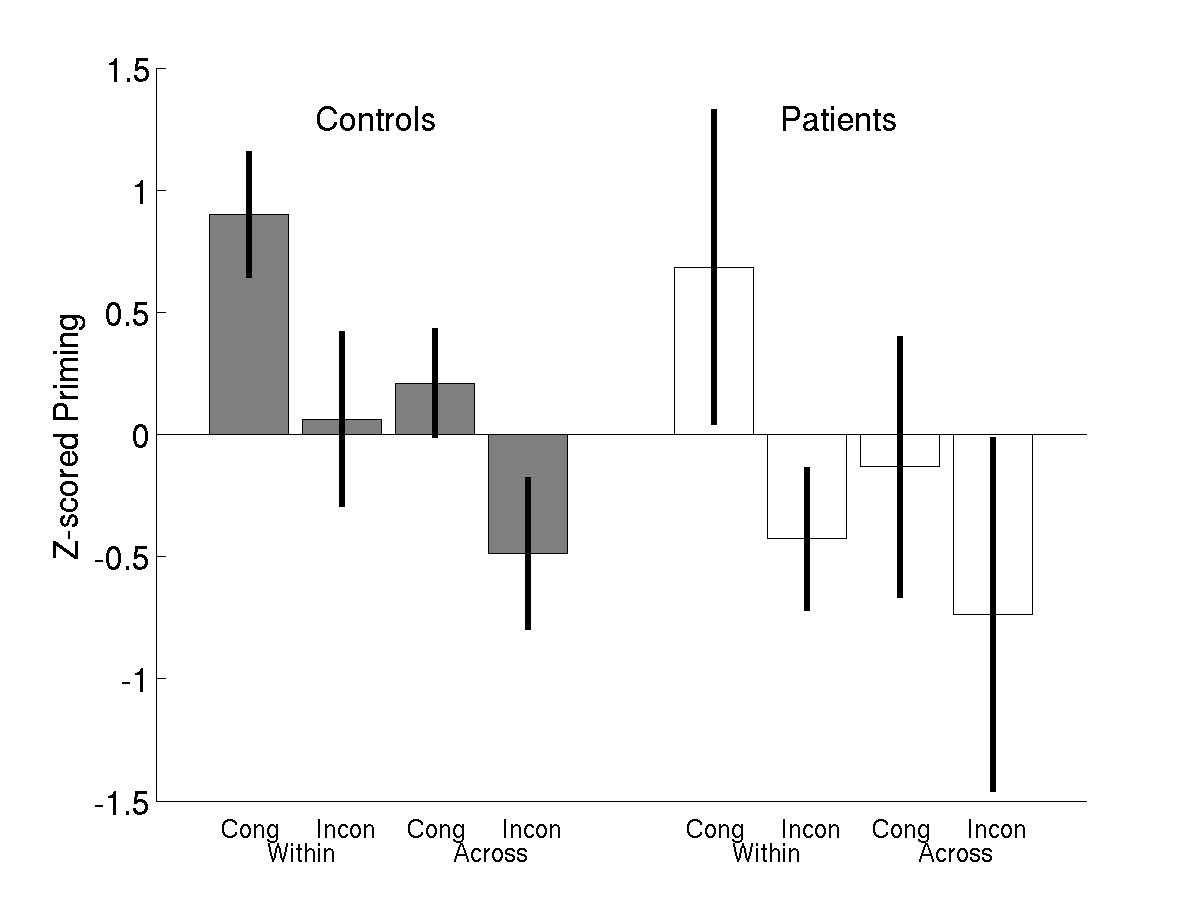


Supplementary Figure 1: Z-scored Priming for each condition and group. Cong=Congruent; Incon = Incongruent. Error bars are one-tailed 95% confidence intervals. See Figure 2 in main paper.

## Adjusting for Sex

Given that one of the six patients were female, yet 13 of the 24 controls were female, the main analyses were repeated with sex as a covariate.

#### Error Analyses

The 2x2x2 ANOVA on subtractive priming of errors showed no significant effects or interactions between Format Match, Congruency and Group, F(1,27)’s < 1.87, p’s > .18, matching the pattern without adjusting for sex in the main paper.

#### RT Analyses

The 2x2x2 ANOVA on proportional priming showed a significant main effect of Congruency, F(1,27)=13.7, p<.001, with positive priming (response speeding) for Congruent conditions and negative priming (response slowing) for Incongruent conditions, together with a significant main effect of Format Match, F(1,27)=23.1, p<.001, with more positive priming within formats rather than across formats, as expected. The interaction between Congruency and Format Match did not reach significance, F(1,27)<1. The main effect of Participant Group was not significant, F(1,27)=2.24, p=.146, and nor was there evidence for an interaction between Participant Group and Congruency, F(1,27)<1, nor between Participant Group and Format Match, F(1,27)<1, nor three-way interaction, F(1,27)=1.19, p=.177. Thus the only change after covarying out sex was that the main effect of participant group was no longer significant, but this only bolsters the general claim of the present study that patients perform similarly to controls.

For subtractive priming, the same 2x2x2 ANOVA on subtractive priming showed a significant main effect of Congruency, F(1,27)=5.81, p<.05, together with a significant main effect of Format Match, F(1,27)=13.0, p<.001, and significant main effect of Group, F(1,27)=6.52, p<.05. Again, any interaction between Congruency and Format Match did not reach significance; nor did the three-way interaction between Congruency, Format Match and Group, F(1,27)’s<1. The two-way interactions between Group and Congruency, F(1,27)=3.71, p=.065, and between Group and Format Match, F(1,27)=4.53, p<.05, approached or reached significance, unlike the results for proportional priming, but like for the analysis of subtractive priming without adjustment for sex. Again though, these interactions reflected a larger effect of Congruency and of Format Match on subtractive priming for Patients than for Controls, i.e, Patients were actually *more* sensitive to S-R effects than were Controls (if slower RTs overall do not matter).

## Excluding P3 with long RTs

Patient P3 had particularly long RTs in all conditions, so in case this patient was using different cognitive processes, we repeated the analysis of proportional priming without this patient. The 2x2x2 ANOVA showed a significant main effect of Congruency, F(1,27)=26.8, p<.001, together with a significant main effect of Format Match, F(1,27)=24.7, p<.001, but no significant main effect of Group, F(1,27)=2.88, p=.101. No two-way interaction reached significance, F(1,27)’s<1.6, p’s>.22.

However, the three-way interaction did now reach significance, F(1,27)=6.49, p=.017, unlike the results with Patient P3. This reflected a bigger effect of Congruency on the Within-Format conditions than the Across-Format conditions in the Patient group than in the Control group, as shown in Supplementary Figure 2. Comparison with the priming effects with P3 included (i.e, Figure 2 in main paper) suggests that the main effect of removing P3 was to make priming in the Congruent Across-Format condition more negative. However, the degree of negative priming in this condition did not reach significance in either analysis. Therefore, we did not pursue this three-way interaction further. The more important result for present purposes was that there was still no evidence that the Patient group showed a smaller effect of Congruency than the Control group (e.g., still no significant two-way interaction between Congruency and Group). Indeed, although the numerical size of the mean congruency effect (averaged across Format Match) in the Patient group decreased from M=16.9% to M=9.85% when P3 was excluded, this figure continued to be larger than for Controls (M=9.72%).


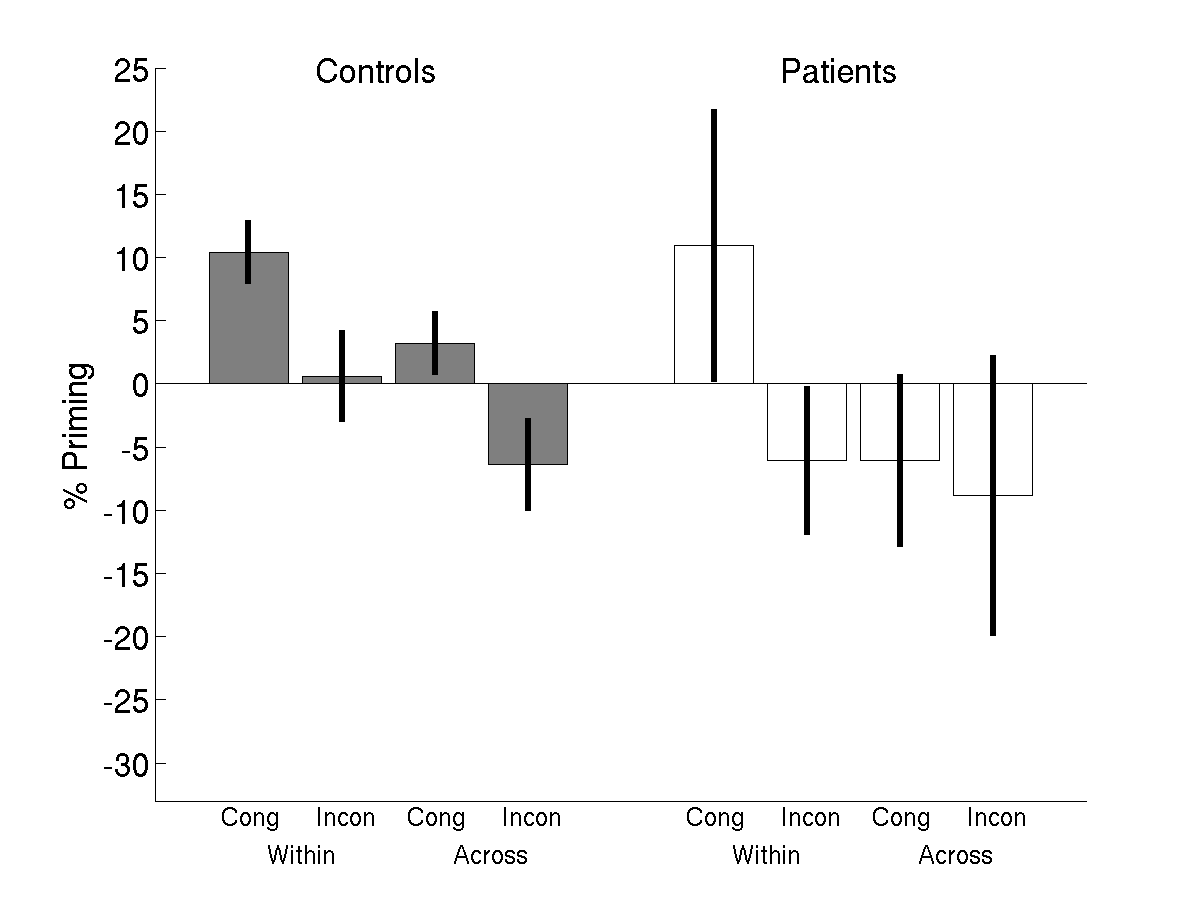


Supplementary Figure 2: Proportional Priming for each condition and group, with Patient P3 removed. Cong=Congruent; Incon = Incongruent. Error bars are one-tailed 95% confidence intervals. See Figure 2 in main paper.

## Excluding P4 with executive impairments

Patient P4 was the only patient significantly impaired on a neuropsychological test other than memory, namely executive function. So in case this patient was using different cognitive processes, we repeated the analysis of proportional priming without this patient. The 2x2x2 ANOVA showed a significant main effect of Congruency, F(1,27)=20.6, p<.001, together with a significant main effect of Format Match, F(1,27)=27.2, p<.001, but no significant main effect of Group, F(1,27)=2.41, p=.132. No interactions reached significance, F(1,27)’s<1.15, p’s>.293.

## Analysis of Within-Format conditions only

Finally, to compare more closely with the Schnyer et al. paradigm, we conducted a 2x2 ANOVA on proportional priming in our Within-Format condition only. The significant main effect of Congruency remained, F(1,28)=18.2, p<.001, but there was no significant main effect of Group, F(1,28)<1. Importantly, any interaction between Congruency and Group did not reach significance, F(1,28)=3.52, p=.07, and the numerical pattern was actually a greater, rather than lesser, effect of Congruency in the Patient group (Figure 2).
